# Exploring the experiences of dancers who have achieved peak performance: on-stage, pre-stage, and post-stage

**DOI:** 10.3389/fpsyg.2024.1392242

**Published:** 2024-05-24

**Authors:** Soo Mi Nam, Hye Youn Park, Min Joo Kim

**Affiliations:** ^1^Division of Sports Science, Hanyang University, Ansan, Republic of Korea; ^2^Institute of Sports Science, Seoul National University, Seoul, Republic of Korea; ^3^Division of Sports and Exercise Science, Kunsan National University, Gunsan-si, Republic of Korea

**Keywords:** performance psychology, peak performance, professional dancer, on-stage, pre-stage, post-stage

## Abstract

The aim of this study is to identify and classify the different attributes that contribute to peak performance among professional dancers, and to understand how these attributes change over time. We conducted an analysis using inductive content analysis on open-ended survey data collected from 42 formally trained professional dancers. Additionally, we analyzed interview data from seven professional dancers who demonstrated outstanding achievements in the field among the survey participants. The main themes that emerged were related to various temporal events of peak performance experience: pre-stage, on-stage, and post-stage. During the on-stage, peak performance was perceived by both internal and external factors. During the pre-stage, emphasis was placed on technical, cognitive, and artistic strategies during practice, whereas just before going on the stage, attention shifted to psychological and physical strategies. During the post-stage, dancers reported immediate changes in their psychological and physical states following the peak performance experience, and thereafter, the peak performance experience was noted to influence psychological, technical, and cognitive aspects. These findings provide valuable insights into the key characteristics that emerge throughout a series of peak performance experiences and are consistent with previous research.

## 1 Introduction

Dance is a unique form of human movement that combines artistic creativity and expression with technical precision and stability of movement. As a performance art, dance is created for audiences to see dance pieces with original choreography and a high level of techniques. Therefore, dancers who perform dance spend much time and effort to showcase their best on stage. When a dancer achieves a flawless performance on stage, it is called a peak performance. Peak performance is a concept that includes not only the excellence of the performance itself but also the perceived subjective experiences of the performer (Privette, 1983; Swann et al., 2017), and in the context of dance performance, peak performance refers to a state in which a dancer performs an outstanding and dominant function, and achieves the highest achievement result by performing an optimal dance performance beyond the limits of his or her function (Flower, 2019). Outstanding dancers experience positive psychological states such as flow and optimal experience when they actually demonstrate peak performance (Hefferon and Ollis, 2006). This represents a state of fully functioning potential for the dancer, and it also allows them to discover their creative self and achieve outstanding performance (Jackson and Roberts, 1992). The struggle of dancers and coaches to reach and maintain this peak performance continues in the field and research. Research on proficiency in dance has primarily focused on the kinematic analysis of ballet, where the kinematic and kinetic variables of essential movements in ballet, such as the Grand rond de Jambe (Wilson et al., 2004), Pirouette (Lin et al., 2019), and Arabesque (Bronner, 2012) have been studied. These findings have been used to identify and improve dancers’ skill levels.

Recently, there has been a growing body of research on the excellence of professional dancers, exploring various aspects such as the relationship between the segmentation of dance movements and expertise in dance (Bläsing, 2015), as well as the impact of observers’ physical abilities on the aesthetic evaluation of dance (Cross et al., 2011). Compared to sports, where peak performance is one of the most studied areas, the study of dance proficiency seems to have received less attention. In sports, where peak performance has been studied extensively, the characteristics of a skilled athlete or player have been characterized by physique and fitness (Tsolakis and Vagenas, 2010), kinematic characteristics of motor skills (Camomilla et al., 2018; Oliveira et al., 2020), kinematic characteristics (Szafrański and Boguszewski, 2015), and cognitive traits such as visual navigation (Brams et al., 2019; Silva et al., 2022) and reaction time (Tønnessen et al., 2013). In addition, studies on the psychological states of skilled athletes and psychological skill training during peak performance moments have been actively studied in the context of peak performance (Ungerleider, 2005), and recently, neuroscience has also been investigating the neurological characteristics of skilled and unskilled athletes (Wei et al., 2011; Kim et al., 2014). On the other hand, in the field of dance, research has been mainly conducted in terms of analyzing the technical aspects of dance movements as described above, and it seems that it is necessary to apply the concept and understanding of peak performance to the field of dance in order to expand the research on dance proficiency. Professional dancers who achieve proficiency under a rigorous training regime can be considered both performing artists and elite athletes (Hays, 2002). Thus, to gain a deeper understanding of dance proficiency, this study aims to investigate the process of peak performance experience among professional dancers who are actively involved in national dance companies.

Approaches to peak performance (Privette, 1981) have been developed under a variety of terms, including peak experience (Maslow, 1962), flow (Csikszentmihalyi, 1990), and aesthetic experience (Sandelands and Buckner, 1989). Maslow (1962) found that prominent figures such as Albert Einstein, Eleanor Roosevelt, and Albert Schweitzer all had in common that they reported mystical experiences in their lives that had great rapture and meaning, and he referred to these moments of pure, positive happiness as peak experiences. Csikszentmihalyi (1990) found that people felt an exhilarating sense of transcendence and a distortion of time and space when they were at the peak of their abilities, which he called the optimal experience and referred to as the state of consciousness associated with flow. Sandelands and Buckner (1989) explained that active participation in an art-like task can lead to an aesthetic experience, considered peak performance. As can be seen from the various definitions, when performance, and thus the performer’s perceived experience, is at its peak, the performer seems to perceive his or her competence as exceptionally good. Privette (1981), who also defined excellence in performance with the word peak performance, stated that peak performance is behavior that exceeds or exceeds what is generally expected from an individual’s potential in a given situation. In the field of sports, the psychological characteristics of peak performance have been studied (Privette, 1983; Williams and Krane, 2021), peak performance strategies have been prepared based on the results (Harmison, 2006), and various psychological skills training have been applied in the field (Ungerleider, 2005) to attract and maintain athletes’ performance at the optimal level. According to previous research, the ideal state of mind and body associated with peak performance is (1) high confidence and anticipation, (2) energized but relaxed, (3) in control, (4) entirely focused, (5) intense focus on the target task, (6) positive attitudes and thoughts about performance, and (7) firm determination and enthusiasm (Williams and Krane, 2021). In a sporting context, peak performance refers to the moment of excellence that occurs when an athlete is at their best physically and mentally (Privette and Brundrick, 1991). To achieve this, athletes spend long hours preparing their bodies, engaging in intense training, working tirelessly, and utilizing various psychological techniques to modulate and enhance the positive factors that influence performance to achieve peak performance in competition (Vealey, 2007). Peak performance is most likely to occur when there is a harmonious alignment of physical, technical, and psychological demands, indicating a unified state of mind and body. Peak performance is also known to be influenced by personal factors such as the practice and competition situations experienced by the performer, as well as contextual variables such as social relationships with family, peers, and coaches, and facilities and equipment (Carlson, 1988; Côté, 1999). From these findings, it is known that skilled individuals are differentiated from novices and unskilled individuals by genetics, the acquired environment, and the interaction of the two in a variety of factors that constitute proficiency, including physique, fitness, skill, cognition, and psychology (Starkes and Allard, 1993). Advances in neuroscience are also revealing the neurological characteristics of skilled athletes. Skilled divers have increased cortical thickness (Wei et al., 2011), and elite archers show greater activation of cortical areas related to visuospatial, spatiotemporal, and decision-making compared to unskilled archers just before shooting a bow (Kim et al., 2014). Similarly, in sports and dance, proficiency occurs when all areas of the brain are at their best, not just the motor skill performance.

If we apply these sports-related proficiency studies to dance, we find that, like sports, dance proficiency can be characterized by the dancers’ physique, fitness, technique, cognition, and psychology. Borrowing from these concepts of proficiency, a review of studies on dance proficiency reveals that the following dimensions have been studied: physique (Misigoj-Durakovic et al., 2005), physical strength (Wyon et al., 2002), technique (Twitchett et al., 2009; Imura et al., 2010; Krasnow et al., 2011), cognition (Bläsing et al., 2009; Fagundes et al., 2013; Kawalek and Gobet, 2024), and psychology (Adam et al., 2004; Walker and Nordin-Bates, 2010). In particular, if we look at the research on dance proficiency, we can find a tendency to understand peak performance in terms of technical aspects such as body conditioning and body alignment (Kirkendall and Calabrese, 1983; Franklin, 2003). However, unlike sports, dance is a performing art characterized by presenting a predetermined sequence with the highest skill level in a set location and time. Therefore, peak performance is required to effectively convey messages and evoke emotions from audiences spaced at intervals (Hamilton and Robson, 2006; Moyle, 2019). Additionally, psychological and attentional demands such as emotional expression, presentation, creativity, artistry, communication, and interaction with the audience (Panebianco-Warrens, 2014) may be differentiated from sports performance studies. Therefore, considering the specificity of the art, it is crucial to maintain an optimal psychological state as well as the technique of physical movement of the performer using the limbs (Nordin-Bates, 2012). This aligns with recent research emphasizing the importance of psychological aspects to peak performance in many areas (Taylor and Estanol, 2015). If you imagine a dancer performing on stage, they have already earned the role because they are already at the top of their game regarding physique, strength, technique, and cognition. This is evidence that they have already reached a certain level in most proficiency-related domains. Psychology can significantly impact dancers’ ability to perform at their best in the moment. Dancers’ tension and pressure on stage can be very high (Rodrigues-Krause et al., 2015). Therefore, the dancer must be psychologically prepared to deal with personal and environmental stresses for optimal performance in a dance performance situation.

There has been less research on proficiency and psychology in dance than in other fields. Studies that do consider psychological aspects are often limited to exploring specific dance genres (Twitchett et al., 2009; Flower, 2016; Flower, 2019), and studies that examine technical and holistically psychological aspects are sorely lacking (Anderson et al., 2014; Franklin, 2018). Furthermore, the time before and after a dancer performs on stage is also a significant factor influencing dance performance, but the literature is sparse. The psychological state of a dancer can be categorized into different stages based on the actual performance on stage. Dancers undergo rigorous training and consistent practice in preparation for performances. Before taking the stage, they go through processes such as makeup, costume selection, and warming up. On stage, dancers have to consider many factors that affect their performance: environmental factors such as the stage, audience, costumes, and weather, as well as personal factors such as physical fitness, technique, and psychology, in order to maintain an optimal state of mind to perform at their best (Sanders et al., 2021). The uniqueness of performing on stage is an essential factor contributing to the differences between performance in practice and training situations (Jaque et al., 2020), and psychological preparation is an integral part of this process, resulting in some performers performing worse or better on stage than in practice. This difference clearly distinguishes between practice and actual performance outcomes and can be interpreted as a significant psychological factor influencing Dancers’ peak performance on stage, in addition to the factors influenced by practice and training. Therefore, to systematically understand why and how peak performance is experienced, a systematic exploration of the psychological states experienced by dancers before, during, and after the main performance and the process of peak performance should be considered. In this study, we aimed to classify the psychological states experienced by professional dancers before, during, and after their performances according to the time series flow and to identify the characteristics of each time point.

## 2 Methods

This study consisted of an open-ended questionnaire and interviews. Open-ended questionnaires encourage participants to freely express their experiences, views, thoughts, and feelings and are an easy way to collect comprehensive data from many participants. At the same time, interviews allow for an in-depth understanding of peak performance experienced by professional dancers through profound conversations (Wittenberg et al., 2013). Peak performance is not a simple, one-dimensional state that can be easily attained (Harmison, 2006). Therefore, through comprehensive data collection using open-ended questionnaires and more specific and in-depth empirical exploration through interviews, we sought better to understand the nature of successful performance in dance and holistically examine the processes involved in peak performance to explain this complex relationship.

### 2.1 Participants

A total of 42 professional dancers (female = 39, male = 3) currently affiliated with a national or public professional dance corps in Korea and with more than 5 years of activity experience participated voluntarily in the study. The average age of the dancers was 38.8 years (*SD* = 5.2 years), and the average dancing experience was 10.8 years (*SD* = 4.6 years). All dancers had formal training in both classical and contemporary dance. Among the dancers who participated in the open-ended survey, seven professional dancers with excellent prize-winning performances and high positions were interviewed (Table 1). Ethical approval for the study was granted by the Institutional Review Board of Seoul National University (IRB NO. 2402/001–023).

**Table 1:** Characteristics of dancers.

Interviewee	Workplace	Career (years)	Age (years)
Dancer 1	Municipal dance company	15	38
Dancer 2	Provincial dance company	14	38
Dancer 3	Municipal dance company	13	38
Dancer 4	Municipal dance company	12	39
Dancer 5	Private dance company	12	38
Dancer 6	National dance company	10	38
Dancer 7	Private dance company	10	38

### 2.2 Procedure

To develop survey items for an open-ended survey, a panel of four experts (including one sports psychology researcher, one sports psychology Ph.D., and two dance Ph.Ds.) convened for a meeting based on the literature review. Through this meeting, semi-structured questions were formulated, focusing on the experiences related to peak performance during pre-, on-, and post-stage. Initially formulated questions underwent pilot testing with two professional dancers and were subsequently revised and refined. The research results did not include the data collected during the pilot test. The detailed questions of the open-ended questionnaire, consisting of a total of seven items, are as follows: (1) When you had your peak performance, what, if any, were the salient features different from your usual performance? (2) When you experienced your peak performance, what preparation enabled you to dance your best? (3) What preparation did you do, especially right before you went on stage? (4) How did you feel immediately after your peak performance? (5) How do you think you have changed since experiencing the peak performance compared to before? (6) How often have you had similar experiences since experiencing the peak performance? (7) How do you think the peak performance experience has changed or influenced your dance performance?

**Figure 1:**
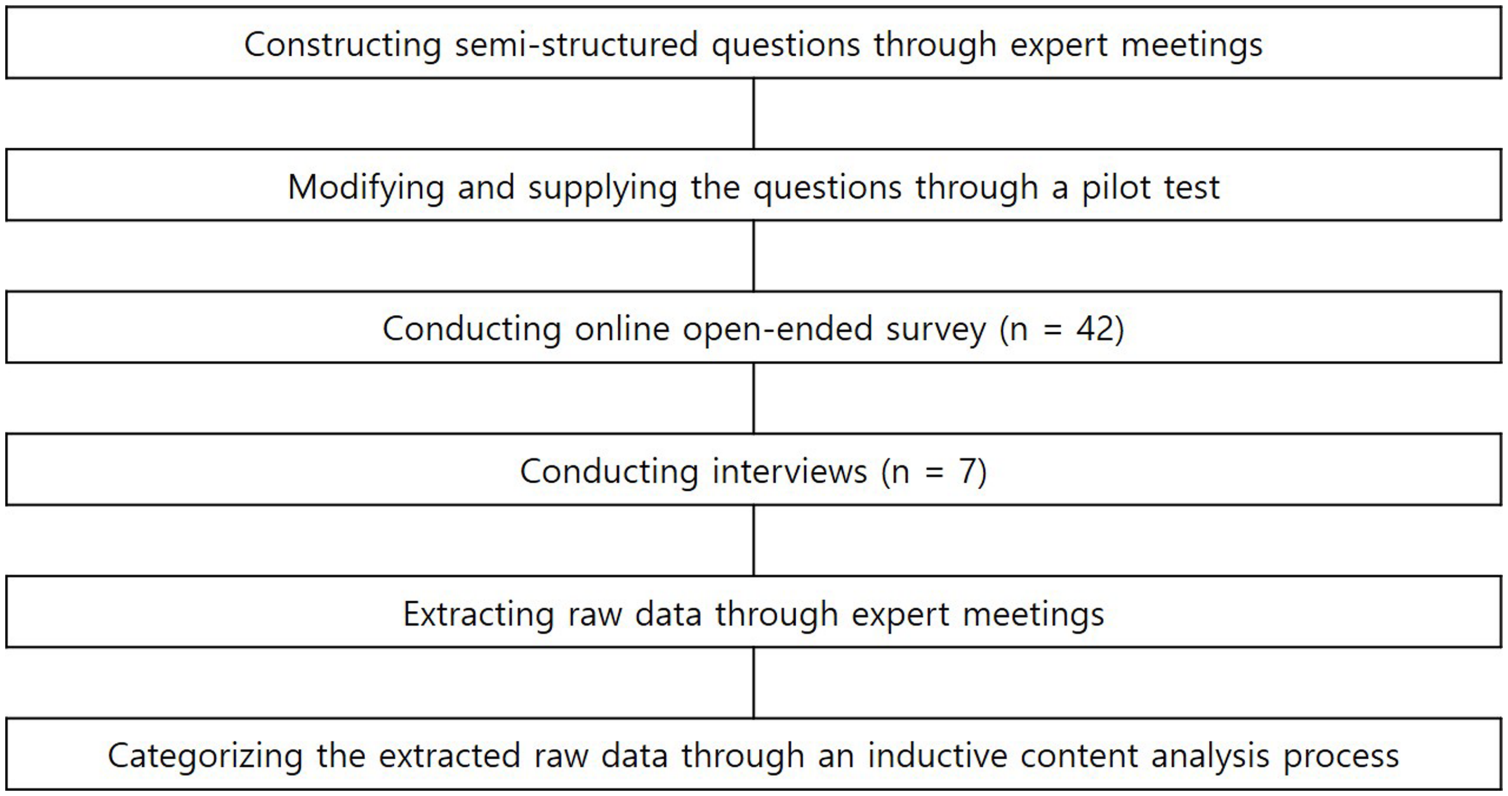
Flow chart for research procedure.

Recruitment of participants for the open-ended questionnaire was facilitated through the personal connections of the first author. Participants were then asked to introduce the study to others with similar characteristics through snowball sampling. Snowball sampling is an acknowledged and effective approach for enlisting participants in a study who may be challenging for the researcher to access or identify (Naderifar et al., 2017). In essence, it is a prevalent sampling technique in qualitative research, wherein the researcher does not directly engage with participants but instead reaches out to individuals who, in turn, facilitate connections with potential research participants (Parker et al., 2019). Only those who expressed their willingness to participate in the study were sent an online link to the mobile app using Google Forms to respond to the questionnaire, and the completed response sheets were collected online. Among the respondents of the open-ended questionnaire, professional dancers with high positions or excellent prizes were selected and interviewed after obtaining their informed consent. Before conducting the interviews, the researcher explained the purpose of the study and the concept of peak performance in dance. Participants were first asked to recall a specific performance experience accompanied by peak performance. This was defined as ‘a state of exceptional and superior functioning, surpassing the limits of one’s abilities to achieve optimal dance performance.’ They were then asked to describe how their preparation before the peak performance affected the peak performance and what changes occurred afterward. The interviews were conducted at a time and place convenient to the dancers. All interviews lasted an average of 40 min and were digitally recorded with the participant’s consent. The interviews utilized a semi-structured format based on the seven questions used in the open-ended survey to obtain additional information or details that may have been missed, and important words were noted (in addition to processing questions about the internet survey). The interviews aimed to obtain a feel for each dancer’s experience and detailed explanations of the factors that affected performance. Based on the collected open-ended questionnaire responses and transcripts of the interviews, raw data was extracted through expert meetings, and the extracted raw data was categorized through an inductive content analysis process. Figure 1 illustrates the research process.

### 2.3 Data analysis

The analysis method used in this study utilized the six-step qualitative analysis method proposed by Creswell (2014). First, the raw data collected through the open-ended questionnaire were excluded if the responses were outside the scope of the question, and if the meaning was ambiguous, the participants were rechecked to clarify the meaning. All recorded data was transcribed by a single researcher for documentation. Second, the raw and final transcribed data from the open-ended questionnaire were read through repeatedly, and key concepts were identified for the first round of categorization. Third, the events were comprehensively delineated within their contextual framework, during which codes were formulated and textual content was systematically categorized. Fourth, qualitative information about the texts was categorized and developed by exploring specific themes or dimensions. Fifth, to interpret the results of the analysis, we sought to explore more significant meanings than in the previous step, and we tried to reach a consensus based on the various interpretations of the researchers. Sixth, we presented and visualized the meaning of the final results of the analyzed data. In addition, the frequencies and percentages of the results derived from the inductive content analysis were calculated for each time point to facilitate an intuitive understanding of the data.

Several methods were applied to ensure the trustworthiness of the data. Each participant received a copy of the interview transcript and was allowed to confirm the accuracy of the content. Member checking was utilized as a means to assess the precision of the transcripts. To minimize data contamination, the researcher who conducted the interviews conducted the transcription and coding, and the transcripts, codes, and categorizations were critically reviewed by peer reviewers.

## 3 Results

The study analyzed the peak performances of professional dancers at three different time points: during the performance, before the performance, and after the performance.

### 3.1 On-stage: peak performance experience of dancers

As a result of conducting an inductive content analysis of professional dancers’ on-stage peak performance experiences, a total of 116 raw data themes were extracted and categorized into six higher-order themes and two categories (Table 2). Specifically, upon closer examination, the general area that exhibited the highest frequency of response was the internal factor, accounting for 67.24%, while the external factor was 32.76%. Among the internal factors, the psychological aspect was the highest at 43.10%, followed by technical (20.69%) and physical (3.45%). Regarding the external factors, the audience accounted for 16.38%, followed by works (11.21%), and music (5.17%). The detailed frequencies are shown in Table 2.

**Table 2:** On-stage: peak performance experience of dancers.

Raw data themes	Higher order themes	Categories
Flow on stage (11) Focus on the internal emotions in work (8) Peace of mind (8) Momentary focus on action (8) Confidence (5) Catharsis for performance (4) Pleasant tension (3) Feel of being in a transcendent space (3)	Psychology (50)	Internal factors (78)
Better performance than practice (6) Automated performance (6) Harmonized performance with dancers (5) Successful Matching perfect timing (4) Breathing control (3)	Skills (24)	
Perception of light-weight body (2) Expansion of body energy (2)	Physical body (4)	
Connection with audience (7) Focus of audience (7) Response of audience (5)	Audience (19)	External factors (38)
Thoroughly exhaustive prepared work (4) Work that suits for me (2) Challenging work (2) Works in which I was given a major role (5)	Works (13)	
Matching movement with music (4) Connecting with musicians (2)	Music (6)	

The numbers within the parentheses denote the frequency of citations.

As evident from the results, the prominent characteristics perceived by professional dancers through peak performance experiences were more focused on the dancers’ intrinsic factors. Most of them described positive psychological experiences during peak performance. In particular, internal factors related to psychology, such as ‘flow on stage,’ ‘momentary focus on action,’ ‘peace of mind,’ and ‘focus on internal emotions in work,’ seem to impact recognizing peak performance significantly.

#### 3.1.1 Internal factors

During peak performance on stage, dancers perceive peak performance through psychological, technical, and physical factors. Dancers reported psychological factors such as ‘flow on stage,’ ‘momentary focus on action,’ and ‘focus on internal emotions in work’ as differing between stages where peak performance was experienced and those where it was not. Additionally, skills factors such as ‘better performance than practice,’ ‘automated performance,’ and ‘harmonized performance with dancers’ were observed. Furthermore, physical body factors such as ‘perception of light-weight body’ and ‘expansion of body energy’ were reported. These factors can be state features that dancers internally felt while performing their peak performance in a specific space, the stage. Based on the inductive content analysis, the primarily mentioned intrinsic psychological factors in the interview content are as follows. The interview content is listed in descending order based on the factors with the highest frequency.

When everything fell into place, such as when I had great chemistry with my fellow performers and achieved harmony with the crew, including dancers, stage crew, and musicians, I felt the most comfortable dancing on stage. Even the environment, like the stage floor, contributed to this feeling. It was during these moments that I could immerse myself fully and dance with the utmost satisfaction.

Dancer 2

I immersed myself in and focused solely on what I needed to do. Consequently, I didn’t pay attention to unnecessary things. My focus was on the aspects I needed to uphold—like gaze, footwork, movements, breathing, body direction, rhythm, and sensation—essential for dancing. Therefore, I concentrated solely on those aspects.

Dancer 6

Even though we practice the same way every time, we can’t always evoke emotions. We can’t cry every time, and it’s impossible to feel the same emotions consistently. However, when experiencing peak performance, emotions are fully immersed, and the desired emotions come naturally. I executed the emotions just as I had practiced, meticulously considering each detail.

Dancer 4

#### 3.1.2 External factors

In addition to the internal factors described above, dancers also perceived peak performance through external factors such as the audience, music, and work. Specific areas of external factors reported by dancers included audience factors, such as ‘connection with audience,’ and ‘focus of audience’; music factors, such as ‘matching movement with music’; and work factors, such as ‘works in which I was given a major role’ and ‘thoroughly exhaustive prepared work.’ These factors can be considered as external experiential characteristics that dancers perceive from the external environment when they exhibit peak performance. Upon conducting inductive content analysis, the prominently mentioned extrinsic factors related to the audience in the interview content are as follows:

I felt like I was breathing together with the audience on stage. Since it was a confident performance, there was a sense of communication and empathy with the audience, as well as the attention directed towards me. Moreover, the response after the performance was also very positive.

Dancer 6

The audience’s response is very enthusiastic; there are applause and cheering, and sometimes even shouts of ‘bravo’. After the performance, when we meet the audience, their facial expressions change. Their undeniable satisfaction is evident. You can tell from their expressions whether they found it boring or enjoyed the performance.

Dancer 7

It felt like I became the protagonist in a TV show during the peak performance. It was as if the audience was solely focused on me. It felt like I was the only one on that stage, just like how we watch the protagonist on TV.

Dancer 5

### 3.2 Pre-stage: preparing for peak performance

To explore what preparation is needed for peak performance, an inductive content analysis was conducted, and 200 raw data were extracted and categorized into five higher-order themes and two categories (Table 3). In the categories, the factor with the highest response frequency was during practice (55%), followed by just before going on stage (45%). Among the subcategories during practice, technical strategies (29%) were the most prominently mentioned, followed by cognitive strategies (15%) and artistic strategies (11%). Regarding just before going on stage, psychological strategies were the highest at 29%, with physical strategies following at 16%.

**Table 3:** Pre-stage: preparing for peak performance.

Raw data themes	Higher order themes	Categories
Practicing steadily (16) Practicing enough (16) Automating dance performance (8) Training with lessons (5) Participating in supplemental physical training (pilates, yoga, personal training, etc.) (5) Training like the actual stage (4) Refining technique repeatedly for perfection (4)	Technical strategies (58)	During practice (110)
Focusing on the dance (12) Keeping persistent imagery of the stage (5) Understanding art work sufficiently (5) Utilizing different ways of focus (4) Conducting objective self-evaluating after training (4)	Cognitive strategies (30)	
Empathizing with characters (10) Training with different emotions (4) Choosing works and characters that work well with you (4) Using expressions to make your actions stand out (2) Analyzing character intensively (2)	Artistic strategies (22)	
Controlling mind in stable status (18) Emptying own mind and heart (10) Taking time alone to focus on the stage adaptation (6) Following own routine (5) Keeping the enjoying mindset (5) Training images of specific movements (5) Self-talking for confidence (5) Believing in oneself (5) Taking a deep breath (4) Having a firm willing for a successful stage (3) Having meditation (3)	Psychological strategies (58)	Right before going on the stage (90)
Taking a warm-up sufficiently and properly (15), Staying in fine-tuned physical condition (12) Practicing specific movement (5)	Physical strategies (32)	

The numbers within the parentheses denote the frequency of citations.

The results showed that the focus was primarily on technical, artistic, and cognitive strategies during practice. Particularly, technical strategies predominated, with most dancers emphasizing ‘practicing steadily’ and ‘practicing enough.’ However, psychological and physical strategies appeared predominantly utilized just before stepping onto the stage. Specifically, psychological techniques such as ‘controlled mind in stable status,’ ‘Emptying own mind and heart,’ and ‘taking time alone for focus on the stage adaptation’ were frequently mentioned, aiding in maintaining a stable psychological state.

#### 3.2.1 During practice

When we looked at what preparation before the performance helped the dancers achieve peak performance, we found that technical, artistic, and cognitive strategies had the most impact in that order. The technical strategy used by the dancers during practice included ‘practicing steadily,’ ‘practicing enough,’ and ‘automating dance performance.’ In contrast, the artistic strategies included ‘empathizing with the character,’ ‘training with different emotions,’ and ‘choosing works and characters that works well with me,’ and the cognitive strategies included ‘focusing on the dance,’ ‘Keeping persistent imagery of the stage,’ and ‘understanding art work sufficiently.’ Here are some of the most frequently mentioned technical strategies in the interviews that emerged from the inductive content analysis.

When I encounter areas of dissatisfaction or receive continuous feedback from others, or if there’s repetitive discussion about certain aspects, I tend to focus intensively on practicing those areas alone. Sometimes, I also seek assistance from others through lessons to improve. Nevertheless, the ultimate solution lies in repetitive practice. After all, there’s a set time frame until I go on stage, so I must give my best within that time frame to avoid regrets.

Dancer 7

I practice a lot, prepare thoroughly, and focus on what I need to do accurately. I concentrate consciously and pay attention to each movement sharply. I practice until I become familiar with the sequence of dance movements and they stick to my body, checking my reflection in the mirror. I practice until my body moves naturally, smoothly, and comfortably, without any buffering, as easily as water flows.

Dancer 6

#### 3.2.2 Right before going on the stage

On the day of the performance, just before taking the stage, dancers reported utilizing both psychological and physical strategies. Psychological strategies were more commonly utilized than physical strategies. Some of the psychological strategies used by dancers included ‘steady mind control,’ ‘clearing my mind and thoughts,’ and ‘getting alone time to adjust to the stage,’ while physical strategies included ‘warming up’ and ‘being in the best physical condition.’ Some of the most common psychological pre-stage strategies emerged from the inductive content analysis.

When I was young, I used to stretch my body a lot, but as I got older, it became more difficult for my body. I felt more tired before going on stage. So, it varies depending on the individual and the extent of injuries, but as I gained more experience, I found that conserving energy and focusing more on mind control after adequately stretching my body seemed to work better for me.

Dancer 4

Before going on stage, I warm up my body and showcase what I’ve been practicing all along. Rather than dwelling on things that didn’t go well, I try to empty my mind and relax my body and mind to relieve tension.

Dancer 3

I don’t do anything specific. Instead, I try to keep it simple and clear my mind. Rather than overcomplicating things, I focus on expressing how I feel in the moment. I just let go and try not to think too much while keeping my mind clear.

Dancer 2

Just before going on stage, I tend to reduce my talking. It’s my time alone. I simply prefer to minimize talking. After that, I follow my own routine.

Dancer 4

It seems important to manage energy more effectively than during practice because more energy is needed physically. To prevent energy from being dispersed elsewhere, I minimize movement in the dressing room and avoid engaging in conversations or distractions. I focus on quietly loosening up my body.

Dancer 2

### 3.3 Post-stage: the impacts of peak performance

As a result of conducting an inductive content analysis on the impact of peak performance on professional dancers, a total of 183 raw data were extracted and categorized into five higher-order themes and two categories (Table 4). First, the category is divided based on the timing at the end of the peak performance and after the peak performance ends. The factor with the highest response frequency in the categories is after the peak performance ended, accounting for 60.66%, followed by right at the end of the peak performance, which accounted for 39.34%. Within the subfield right at the end of the peak performance, psychological state factors showed the highest response frequency at 34.43%, followed by physical state factors at 4.92%. In the subcategories, after the peak performance ended, psychological changes were the most prominent at 26.78%, technical changes at 22.40%, and cognitive changes at 11.48%.

**Table 4:** Post-stage: the impact of peak performance.

Raw data themes	Higher order themes	Categories
Fulfillment (15) Euphoria (13) Jubilation (12) Afterglow (8) Excitement (5) Catharsis (4) Vibrancy (2) Relief (2) Futility (2)	Psychological state (63)	Right at the end of the peak performance (72)
Refreshment (6) Feel of adrenaline being released (3)	Physical condition (9)	
Increased desire to re-experience peak performance (12) Enhanced confidence in dance (12) Improved passion and conviction about dance (8) Enhanced faith regarding one’s own ability (7) Reduced in fear on stage (7) Increased commitment to a successful performance (3)	Psychological changes (49)	After the peak performance ended (111)
Improvement of dancing skills (12) Practice for a more perfect stage (11) Use various expression methods (7) Preparation for the next stage thoroughly (6) Challenges to re-experience peak performance (5)	Technological change (41)	
Expansion of thinking on dance (10) Positive reviews of one’s own dance (7) New discoveries about dance (4)	Cognitive change (21)	

The numbers within the parentheses denote the frequency of citations.

The results showed that psychological reports predominated immediately after the peak performance ended. Right after the peak performance, positive psychological experiences such as ‘fulfillment’, ‘euphoria,’ and ‘jubilation’ were predominant, while afterward, main psychological changes included factors like ‘increased desire to re-experience peak performance,’ ‘enhanced confidence in dance,’ and ‘improved passion and conviction about dance.’

#### 3.3.1 Right at the end of the peak performance

Dancers who experienced peak performance reported their psychological and physical states immediately afterward. Dancers primarily reported on their psychological state, with words like as ‘fulfillment’, ‘euphoria,’ and ‘Jubilation’ dominating the list. They reported feeling ‘refreshment’ and a ‘feeling of adrenaline being released’ regarding their physical condition. Upon conducting inductive content analysis, the prominently mentioned interview content regarding the psychological states immediately felt after experiencing peak performance is as follows:

After putting in the effort to practice diligently and showcasing my abilities on stage, I gained confidence knowing that I’ve performed to the best of my ability. When I come off the stage, I feel a sense of pride, realizing that my skills have improved and that others acknowledge my success. It’s a feeling of accomplishment like I’ve taken a step forward.

Dancer 3

It’s moments like these that make me realize why I dance. The response from the audience, the satisfaction from my performance - that’s why I dance. I feel a sense of joy every day as I hone my skills. Ultimately, I believe we live to find happiness, and experiencing joy as a human being is something I cherish deeply. That’s why I feel like I’ve found my reason for dancing.

Dancer 3

It’s just that I surpassed my limits, and it’s like I experienced something new today, a feeling of discovering something I hadn’t felt before. I was just carried away by that feeling. I felt really excited and happy because I think I discovered a new path in dance that I hadn’t explored before.

Dancer 1

#### 3.3.2 After the peak performance ended

Psychological changes that occurred after peak performance included ‘increased desire to re-experience peak performance,’ ‘enhanced confidence in dance,’ and ‘improved passion and conviction about dance.’ Regarding technical changes, frequent mentions included ‘improvement of dance skills’ and ‘practice for a more perfect stage’. As for cognitive changes, there were reports of ‘expansion of thinking on dance’ and ‘positive reviews of one’s own dance.’ Upon conducting inductive content analysis, the prominently mentioned interview content regarding psychological changes felt after experiencing peak performance is as follows:

It was a stage where I felt like I was shining. I had the experience of wearing clothes that suited me perfectly and shining. (…) I felt proud. I still live on, reminiscing about meeting that stage again. I want to experience it again, showcase it, do better, and do it anew.

Dancer 4

Afterward, I gained a bit more confidence, and actually when I stood on stage, I realized how I could satisfy myself. I focused more on how to practice, how much expression I needed to show, and things like that. So, when I stood on stage, I felt a bit more confident and satisfied because I practiced those aspects more attentively.

Dancer 2

It definitely feels like there was a change. Even in the same dance, there were different forms or movements that I wasn’t used to, but I confidently embraced them as opportunities. When learning something new, I used to feel anxious and wonder if I could really do it, but now those thoughts and worries have diminished. I feel like I can do it all, I have gained a lot of confidence in accepting and being able to do new things.

Dancer 5

If you’re a dancer, you’ve probably experienced all of these things, so I think you’ll keep dancing. Experiences like these are what make you enjoy dancing and keep you going.

Dancer 3

## 4 Discussion

This study aimed to explore the overall process of peak performance as experienced by professional dancers on stage. Collectively, we analyzed data collected through in-depth interviews and an open-ended questionnaire to explore the characteristics of peak performance experiences over time, including the moment of peak performance and the time before and after that moment. As a result, we were able to explore the peak performance experience of professional dancers in three distinct changes over time: on-stage (the moment of peak performance), pre-stage (the preparation for peak performance), and post-stage (the impact of peak performance). Specific discussions for each point in time include. The current findings support previous research indicating that dancers’ success and development are influenced by factors such as motivation, optimal learning, productive thinking, high-level skills, and self-efficacy (Chua, 2014). The details of this elaboration are provided below.

First, a discussion of peak performance moments on stage is presented as follows. The factors that led to professional dancers’ perception of peak performance on stage were broadly categorized into internal and external factors. Dancers commented on the psychological, technical, and physical characteristics they experienced internally during their peak performance. Most professional dancers used words to describe their peak performance experience, implying that it was an extraordinary peak performance that pushed them beyond their limits, along with adverbs to emphasize that it was different than usual (really, more, extremely, and tremendously). These words are categorized as psychological (flow, focus, fulfillment, and catharsis), technical (perfection, success, automaticity, and harmony), and physical (lightness and energy) and represent the state of mind during peak performance. Dancers seem to perceive peak performance in a positive psychological, technical, and physical optimal state on stage. In other words, peak performance can be considered as achieved when there is simultaneous fulfillment of physical, technical, and psychological demands. A study that qualitatively explored ballet dancers’ experiences of peak performance reported that peak performance is characterized by a heightened sense of multiple positive emotions (elation, joy, and fun), an altered state of consciousness (flow), less self-awareness, and an individual’s ability to master technique (Flower, 2016). For a dancer to achieve the highest level of performance, mental preparation, accurate and well-learned technical skills, and a high level of physical health are essential (Taylor and Estanol, 2015). Moreover, the attainment of improved flow states plays a vital role in achieving successful performance optimization (Kirchner et al., 2008; Jaque et al., 2020). Also, the characteristics of the flow state closely align with those of peak performance (Jackson et al., 2001; Hefferon and Ollis, 2006). In the context of research on dancers’ flow, the significance of maintaining a balance between the performer’s challenge and skill is emphasized, along with the ability to sustain effortless long-term attention and focus while executing the task (Jaque et al., 2020; Thomson and Jaque, 2023). However, flow differs from peak performance in that it is an intrinsically rewarding experience that may or may not result in peak performance (Privette and Brundrick, 1991). The intrinsic factors of peak performance experienced through dance performance that emerged in the present study are also found in studies of peak performance in sports (Ferrell et al., 2006; Harmison, 2006; Williams and Krane, 2021). The peak performance of professional dancers was closely related to the internal factors experienced through dance practice and external environmental factors such as the audience, the work, and the music. Dance performances are executed on a stage in front of audiences, so it is inevitable that in a dance performance, the dancer will convey the message of the piece through peak performance, and the audience will understand and relate to the message as they watch (Atkinson et al., 2004; Nordin-Bates, 2012). This interaction between the dancer and the audience was perceived as an essential factor in the peak performance experience. Dancers reported feeling connected to the audience during peak performance, being sensitive to the audience’s focus or response, and experiencing positive audience reactions. Dancers also mentioned the nature of the work in which they experienced peak performance. Primarily, when they were given a significant role and when the role was challenging or a good fit for them, they said they prepared more thoroughly for the performance, leading them to peak performance. These findings are similar to the motivations that have been found in research related to motivation in sports and performance, where individuals are driven to reach areas of excellence when performing evaluated tasks or participating in competitions (Roberts et al., 2007). In these cases, it is assumed that responsibility for the outcomes of the task and a degree of challenge are inherent (Fry and Moore, 2019). We can see that professional dancers place a high value on being responsible for the outcomes of the task at hand (importance of the role in the work of art), congruence between the requirements of the task, and their personal dance style (homogeneity of the role with their dance), and balance between the level of the task and their abilities (perceived challenge) in their peak performance experiences. These characteristics can be seen as key contextual factors that influence the expression of peak performance.

Second, a discussion of preparation for peak performance on pre-stage is presented as follows. Preparation on pre-stage can be categorized into two distinct time periods: during practice and immediately before the performance. During a given practice period, dancers physically focus on technical, cognitive, and artistic strategies directly related to dance performance to achieve peak performance on stage. Movement proficiency comprises physiological, technical, cognitive, and emotional factors (Williams and Krane, 2021). Skilled dancers spend countless hours training their bodies and honing their skills through systematic and rigorous training and practice to achieve all these components. The more time dancers invest in practice, the greater the likelihood of contributing to improvements in cognitive abilities such as superior perceptual processing and the ability to perform dance tasks automatically (Kawalek and Gobet, 2024). Professional dancers all emphasize the importance of consistent and sufficient repetitive practice. According to them, the right amount and duration of practice is the foundation for a successful performance, and it is also the basis for dancers to be psychologically stable and have faith in themselves before going on stage (Anderson et al., 2014). In addition, during the practice process, they use cognitive methods such as image training, self-assessment, and concentration to maximize the efficiency and effectiveness of practice, analyze and understand the characters in the work, and try to empathize with them to enhance artistic perfection. These various strategies were useful in promoting peak performance, and the most preferred method varied from dancer to dancer. During the pre-stage, it is noteworthy that just before going on-stage, they focused on the psychological and physical aspects rather than the technical, artistic, and cognitive aspects, and this strategy led to peak performance. Dance is one of the performing arts with liveness that cannot be recreated after the moment it is performed. Performing arts with liveness are characterized by one-time performance, in which the acted and choreographed body movements are perceived as a physical reality (object) by the eyes, but after that time, all body movements return to the illusion (Reason and Lindelof, 2016). Considering the performative artistic characteristics of dance, it is efficient to focus on bringing out and maintaining the best psychological and physical conditions of the performers (i.e., dancers), in order to successfully perform the dance performance that has been painstakingly created during the practice period on the day of the performance. Moreover, it is important to implement psychological techniques to manage anxiety stemming from unavoidable or heightened demands experienced during the rehearsal process on the day of the performance (Thomson and Jaque, 2023). In particular, professional dancers have been shown to utilize various psychological techniques elite athletes use in competition, including mind control, magnetization, imagery training, and routines to create an optimal mental state (Ungerleider, 2005; Harmison, 2006). These individualized psychological techniques appear to be important factors in maintaining an optimal mental state and achieving peak performance.

Third, a discussion of the impact of peak performance after the stage is over follows. The post-peak performance experience can be categorized into two distinct time points: immediately after the performance and afterward. Professional dancers reported psychological changes and physiological underpinnings related primarily to dance performance immediately after leaving the stage at the end of a performance. The experience of peak performance on stage led to positive psychological experiences such as feelings of fulfillment, euphoria, and jubilation. Peak performance is a rewarding experience for dancers after repeated practice and hard training. The fulfillment and jubilation experienced through peak performance (i.e., goal attainment) have been shown to lead to secondary psychological changes that trigger and sustain an ‘internal motivation’ to experience peak performance again in subsequent dance performances (Lyubomirsky, 2001; Hacker and Mann, 2022). These findings validate earlier flow studies, affirming that peak performance experiences result in enhanced motivation and positive emotional states, which not only have a direct impact on dancers’ future performances but also expand individuals’ cognitive-behavioral range, thereby improving human achievement and leading to a more satisfying and fulfilling life (Costa et al., 2023). Furthermore, since dance is based on physical movement, sensations such as freshness, exhilaration, and a feeling of warmth throughout the body after sweating can be viewed as vitality stemming from physiological bases. In dance performance, physical vitality and a sense of satisfaction and liveliness after focusing on performance were evident. The immediate post-peak performance state exhibited by dancers is believed to stem from the intrinsic nature of dance, which distinguishes it from other physical activities as an expressive activity that combines the body and mind (Hanna, 1995). The peak performance experience is brief and transient but has lasting and profound consequences afterward. Professional dancers reported psychological, technical, and cognitive changes after their peak performance experience. Specifically, psychologically, professional dancers reported a solid desire to re-experience their peak performance, increased confidence, passion, conviction, and trust in their dance abilities, and decreased fear on stage. Psychological factors change technical dimensions or aspects, such as practicing and thoroughly preparing for more flawless performance and improving dance skills. As a result, the peak performance experience served as a powerful intrinsic motivator for the individual dancer’s positive evaluation, endurance of dance activities, and efforts to improve performance (Privette, 1983; Rheinberg, 2020). It was also noted that the peak performance experience led to a cognitive expansion of thinking about dance, an increase in the diversity of expressive techniques, and a qualitative change in the efficiency or direction of practice.

This virtuous cycle shows that the dancers’ peak performance experience is a major turning point. In other words, the peak performance experience can be interpreted as a trigger for self-growth, enabling the dancer to discover and realize new things and acting as a catalyst for psychological, technical, and cognitive changes related to dance. Although this study cannot establish a causal relationship between the findings, it is still valuable as it offers insight into the psychological factors that professional dancers experience during their peak performances. In the future, studies should be conducted to investigate these causal differences thoroughly.

## Data availability statement

The original contributions presented in the study are included in the article/supplementary material, further inquiries can be directed to the corresponding authors.

## Ethics statement

The studies involving humans were approved by the Institutional Review Board of Seoul National University. The studies were conducted in accordance with the local legislation and institutional requirements. The participants provided their written informed consent to participate in this study. Written informed consent was obtained from the individual(s) for the publication of any potentially identifiable images or data included in this article.

## Author contributions

SN: Writing – original draft, Writing – review & editing, Formal analysis, Methodology, Visualization, Conceptualization, Data curation, Investigation, Resources. HP: Data curation, Project administration, Supervision, Validation, Writing – review & editing, Conceptualization, Investigation, Resources, Writing – original draft. MK: Project administration, Supervision, Validation, Writing – review & editing, Conceptualization, Data curation, Investigation, Resources, Writing – original draft.
